# Three-year multicenter surveillance of community-acquired *listeria monocytogenes *meningitis in adults

**DOI:** 10.1186/1471-2334-10-324

**Published:** 2010-11-11

**Authors:** Rosario Amaya-Villar, Emilio García-Cabrera, Elena Sulleiro-Igual, Pedro Fernández-Viladrich, Dionisi Fontanals-Aymerich, Pilar Catalán-Alonso, Carlos Rodrigo-Gonzalo de Liria, Ana Coloma-Conde, Fabio Grill-Díaz, Antonio Guerrero-Espejo, Jerónimo Pachón, Guillén Prats-Pastor

**Affiliations:** 1 Intensive Care Unit. Hospital Universitario Virgen del Rocío. Av Manuel Siurot s/n, 41013 Sevilla, Spain; 2 Spanish Network for Research in Infectious Disease (REIPI). Hospital Universitario Virgen del Rocío. Av Manuel Siurot s/n, 41013 Sevilla, Spain; 3 Instituto de Biomedicina de Sevilla (IBIS) Hospital Universitario Virgen del Rocío/CSIC/Universidad de Sevilla, 41013, Sevilla, Spain; 4 Clinical Microbiology Department. Hospital Universitari Vall d'Hebron, Av del Valle de Hebron 119-129, 08035 Barcelona, Spain; 5 Infectious Disease Service. Hospial Universitari de Bellvitge, Feixa Larga s/n, 08907 L'Hospitalet de Llobregat, Spain; 6 Clinical Microbiology Department. Corporació Sanitària Parc Tauli, Parc Tauli s/n 080208 Sabadell, Spain; 7 Department of Clinical Microbiology and Infectious Diseases. Hospital Universitario Gregorio Marañón, Doctor Esquerdo 46, 28007 Madrid, Spain; 8 Pediatrics Department. Infectious Disease Unit. Hospital Germans Trias i Pujol, Carretera del Canyet s/n, 08916 Badalona, Spain; 9 Infectious Disease Service. Hospital de la Santa Creu i Sant Pau, C/Sant Antoni Maria Claret, 167, 08025 Barcelona, Spain; 10 Intensive Care Unit Infectious Disease Service. Hospital Ramón y Cajal, Ctra Colmenar Viejo Km 9,1, 28034 Madrid, Spain; 11 Internal Medicine Departament. Hospital de la Ribera, Ctra. Corbera Km.1. 46600 Alzira, Valencia, Spain; 12 Infectious Disease, Microbiology and Preventive medicine Clinical Unit. Hospital Universitario Virgen del Rocío, Av Manuel Siurot s/n, 41013 Sevilla, Spain

## Abstract

**Background:**

*Listeria monocytogenes *is the third most frequent cause of bacterial meningitis. The aim of this study is to know the incidence and risk factors associated with development of acute community-acquired *Lm *meningitis in adult patients and to evaluate the clinical features, management, and outcome in this prospective case series.

**Methods:**

A descriptive, prospective, and multicentric study carried out in 9 hospitals in the Spanish Network for Research in Infectious Diseases (REIPI) over a 39-month period. All adults patients admitted to the participating hospitals with the diagnosis of acute community-acquired bacterial meningitis (Ac-ABM) were included in this study. All these cases were diagnosed on the basis of a compatible clinical picture and a positive cerebrospinal fluid (CSF) culture or blood culture. The patients were followed up until death or discharge from hospital.

**Results:**

Two hundred and seventy-eight patients with Ac-ABM were included. Forty-six episodes of *Lm *meningitis were identified in 46 adult patients. In the multivariate analysis only age (OR 1.026; 95% CI 1.00-1.05; p = 0.042), immunosupression (OR 2.520; 95% CI 1.05-6.00; p = 0.037), and CSF/blood glucose ratio (OR 39.42; 95% CI 4.01-387.50; p = 0.002) were independently associated with a *Lm *meningitis. The classic triad of fever, neck stiffness and altered mental status was present in 21 (49%) patients, 32% had focal neurological findings at presentation, 12% presented cerebellum dysfunction, and 9% had seizures. Twenty-nine (68%) patients were immunocompromised. Empirical antimicrobial therapy was intravenous ampicillin for 34 (79%) of 43 patients, in 11 (32%) of them associated to aminoglycosides. Definitive ampicillin plus gentamicin therapy was significantly associated with unfavourable outcome (67% vs 28%; p = 0.024) and a higher mortality (67% vs 32%; p = 0.040).The mortality rate was 28% (12 of 43 patients) and 5 of 31 (16.1%) surviving patients developed adverse clinical outcome.

**Conclusions:**

Elderly or immunocompromised patients, and a higher CSF/blood glucose ratio in patients with Ac-ABM must alert clinicians about *Lm *aetiology. Furthermore, we observed a high incidence of acute community-acquired *Lm *meningitis in adults and the addition of aminoglycosides to treatment should be avoid in order to improve the patients' outcome. Nevertheless, despite developments in intensive care and antimicrobial therapy, this entity is still a serious disease that carries high morbidity and mortality rates.

## Background

*Listeria monocytogenes *(*Lm*) is a Gram-positive, facultative anaerobic bacterium that primarily causes sepsis and meningitis [1] in either immunocompromised or immunocompetent hosts [2,3]. As a foodborne pathogen, it has emerged as a significant public health problem and has caused several epidemics in the United States and Europe. Community-acquired *Lm *meningitis is a serious and life-threatening disease. The estimated incidence is 0.2 cases per 100.000 adults per year in developed countries [1]. *Lm *is the third most frequent cause of community-acquired bacterial meningitis, after *Streptococcus pneumoniae *and *Neisseria meningitidis *aetiologies, due to the vaccine-related decline in *Haemophilus influenzae *type b meningitis [2]. This entity occurs mainly in immunocompromised patients, newborns, and elderly individuals [4], although previously healthy adults can be affected as well [5]. Even with appropriate antibiotic therapy, this entity has a high morbidity and mortality (24%-62%) [2-4,6-8].

In this report, we set out to study the incidence and risk factors associated with development of acute community-acquired *Lm *meningitis in adult patients and to evaluate the clinical features, management, and outcome in this prospective case series.

## Methods

This is a prospective multicenter observational study carried out in 9 Spanish hospitals. All these hospitals are large institutions with teaching accreditation. The study was conducted over a 39-month period. All patients over 14 years old admitted to the participating hospitals with the diagnosis of acute community-acquired bacterial meningitis (Ac-ABM) were included in this study. The Ethical committees approved the study and did not require informed consent from the patient.

One investigator at each hospital prospectively recorded variables of all Ac-ABM in a previously designed database collecting data on patients' demography, clinical antecedents, symptoms and signs at admission, laboratory findings, clinical course, treatment, and outcome. Episodes of Ac-ABM were diagnosed on the basis of compatible clinical picture and at least 1 of the following cerebrospinal fluid (CSF) findings: CSF polymorphonuclear (PMN) pleocytosis [white blood cells (WBC) count >10/μl, 90% neutrophils, hipoglycorrachia <40 mg/dL or CSF/blood glucose ratio <40%, increased protein level >1 g/L, a positive CSF Gram's stain or culture, and/or positive blood cultures. When the CSF Gram's stain and blood and CSF cultures were negative, meningitis of unknown aetiology was recorded. Meningitis was considered to be community-acquired whenever a patient had not been previously treated in a hospital. If the patient had been previously admitted, Ac-ABM was diagnosed whenever the onset of the disease occurred 2 weeks after discharge or after 4 weeks if the patient had undergone surgery [9].

*Lm *meningitis was diagnosed in those patients with Ac-ABM in whom *Lm *was isolated in the CSF, blood culture, or both. Serotyping *Lm *was done using specific antisera, as described elsewhere [10].

Patients were considered immunocompromised if they were receiving immunosuppressive therapy and/or had haematological and solid malignancy, connective tissue disease, diabetes mellitus, alcoholism, asplenia, liver cirrhosis, end-stage renal disease, or HIV infection [11]. Patients were considered elderly when they were 60 years or older.

The classic triad was defined when fever, neck stiffness and change in mental status were present. Hyponatremia was defined as a plasma sodium concentration <135 mmol/L [12]. Focal neurological deficit was diagnosed when aphasia, monoparesis, hemiparesis or cranial nerve palsies were present at admission or during hospitalisation. Cerebellum dysfunction was diagnosed in patients with nystagmus, dysarthria, or limb and gait ataxia.

Time to admission was defined as the appearance of symptoms to admission to the hospital. Patients with impairment of consciousness, neurological deficits, or seizures, underwent cranial computed tomography (CT) before lumbar puncture.

Empiric antibiotic therapy was defined as any parenteral antibiotic capable of crossing the blood-brain barrier, administered for the purpose of treating bacterial meningitis [8]. In the case of *Lm *meningitis, intravenous administration of penicillin, ampicillin, amoxicillin/clavulanic acid, piperacillin/tazobactam, a carbapenem, moxifloxacin or trimethoprim-sulfamethoxazole was considered adequate empirical therapy. During the follow up performance of a brain CT or the administration of adjunctive therapy (corticosteroids and/or phenytoin) were left for the physician in charge to decide.

All patients were followed up until death or hospital discharge. At discharge, all patients underwent a neurological examination, and outcome was measured according to the Glasgow Outcome Scale [13]. A score of 1 on this scale indicates death; a score of 2, a vegetative state; a score of 3, severe disability; a score of 4, moderate disability; and a score of 5, mild or no disability. A favourable outcome was defined as a score of 5, and an unfavourable outcome was defined as a score of 1-4.

For the statistical analysis the SPSS 16.0 software package was used. Univariate analysis of variables from the entire population was performed using unpaired student's t-test for parametric continuous variables after correction for equality of variance (Levene's test), U Mann-Whitney test for non parametric continuous variables, and Pearson's chi-square test or Fisher's exact test for categorical variables. Statistical significance was considered when p <0.05. All p values were two-sided. Furthermore, a multivariate using logistic regression analysis was used to identify independent associations with *Lm *meningitis with results presented as Odds-Ratio (OR) and the 95% confidence intervals (95% CI). Finally, a univariate analysis comparing immunocompromised and immunocompetent patients with *Lm *meningitis was performed as previously indicated.

## Results

Two hundred and seventy-eight patients with Ac-ABM were included in this prospective study. The most common pathogen was *Streptococcus pneumoniae *(Table 1). Forty-six episodes of *Lm *meningitis were identified in 46 patients. Three patients were excluded from the analysis because they were prematurely transferred to another hospital. *Lm *grew from the CSF in 40/43 (93%) cases and from blood culture in 27/43 (63%).

**Table 1:** Aetiologies in 278 episodes of acute community-acquired bacterial meningitis in adults

Organism	n(%)
*Streptococcus pneumoniae*	135 (48.5)
*Neisseria meningitidis*	63 (22.6)
*Listeria monocytogenes*	46 (16.5)
*Haemophilus influenzae*	9 (3.2)
*Escherichia coli*	4 (1.4)
*Streptococcus agalactiae*	4 (1.4)
Others	17 (6.1)

Table 2 shows the comparison between *Lm *meningitis and episodes with other aetiologies. In this univariate analysis those risk factors and clinical characteristics showing differences at admission were: age, immunosupression, previous otitis, focal neurological deficit, cerebellum dysfunction, WBC count CSF and percentage of neutrophils, CSF/blood glucose ratio, and CSF protein levels. However in the multivariate analysis only age (OR 1.026; 95% CI 1.00-1.05; p = 0.042), immunosupression (OR 2.520; 95% CI 1.05-6.00; p = 0.037), and CSF/blood glucose ratio (OR 39.42; 95% CI 4.01-387.50; p = 0.002) were independently associated with *Lm *meningitis.

**Table 2:** Comparison of episodes of acute community-acquired *Lm *meningitis *versus *other aetiologies

	*Listeria monocytogenes (n = 43)*	Other agents (n = 232)	*p*
Age (median ± IQR)	69 ± 30	56 ± 33	0.019

Gender (male)	24 (56) ^d^	128 (55)	0.938

Immunocompromised	29 (67)	86 (37)	<0.001

Previous otitis	1 (2.1)	43 (19.5)	0.005

Fever	39 (91)	186 (84)	0.246

Headache	29 (67)	166 (77)	0.146

Vomiting	20 (46)	132 (57)	0.070

Neck stiffness	30 (70)	167 (72)	0.204

Classic Triad ^a^	21 (49)	90 (39)	0.533

GCS ^b ^at admission (median ± IQR)	13 ± 4	12 ± 4	0.664

Seizures	4 (9)	17 (7)	0.688

Focal neurological deficit	14 (32)	27 (12)	0.022

Cerebellum dysfunction	5 (12)	5 (2)	<0.001

Hyponatremia	19 (44)	60 (26)	0.066

Duration of symptoms in hours (median ± IQR)	70 ± 53	48 ± 53	0.07

WBC count CSF ^c ^(median ± IQR)	550 ± 2480	2860 ± 9380	0.007

CSF neutrophils ( median±IQR)	70 ± 59	90 ± 13	<0.001

CSF/blood glucose (median ± IQR)	0.26 ± 0.24	0.09 ± 0.26	<0.001

Protein level g/L ( median ± IQR)	2.0 ± 1.96	3.68 ± 5.53	<0.001

Mortality	12 (29)	36 (16)	0.059

^ a ^Triad of fever, neck stiffness and change in mental status.

^ b ^GCS: Glasgow Coma Scale.

^c ^CSF: Cerebrospinal Fluid.

^ d ^Results are expressed as n (%) unless otherwise specified

All patients with acute community-acquired *Lm *meningitis were immunocompromised or over 50 years old. The baseline characteristics of these patients and the differences between patients with and without immunocompromise are shown in Table 3. The median age was 69 ± 30 years and 24 of the 43 cases (56%) were male. Twenty-nine (68%) patients were immunocompromised and fourteen (32%) were immunocompetent patients. The nature of immunocompromise were: Twelve (41%) patients were receiving immunosuppressive therapy, 8 (27.5%) had haematological and solid malignancy, 2 (6.8%) with connective tissue disease, 2 (6.8%) with end-stage renal disease and 5 (17.2%) with HIV infection.

**Table 3:** Characteristics of patients with acute community-acquired *Lm *meningitis

Variable ^a^	Patients	Immunocompromised	Immunocompetent	p *
Number of patients	43	29	14	

Age (median ± IQR)	69 ± 30	69.5 ± 32	64.5 ± 23	1

Elderly (60 years or older)	24 (56)	16 (55)	8 (58)	0.903

Antibiotic therapy before admission ^b^	3/43 (7)	2/29 (7)	1/14 (7)	0.950

Median time to admission (hours)	48.5	48	49	0.500

Time to admission > 48 h	22/43 (51)	15/29 (52)	7/14 (50)	0.916

Symptoms at presentation				

Seizures	4/43 (9)	3/29 (10)	1/14 (7)	0.735

Headache	29/43 (67)	17/29 (59)	12/14 (85)	0.076

Vomiting	20/43 (46)	12/29 (41)	8/14 (57)	0.331

Neck stiffness	30/43 (70)	17/29 (62)	12/14 (86)	0.114

Temperature ≥ 38°C	39/43 (91)	26/29 (90)	13/14 (93)	0.735

Glasgow Coma Scale at admission (median ± IQR)	13 ± 3	13 ± 5	13 ± 1	0.279

Classic Triad ^c^	21/43 (49)	11/29 (45)	10/14 (71)	**0.039**

Focal neurological deficit				

Any	14/43 (32)	8/29 (28)	6/14 (43)	0.317

Cranial nerve palsies	7/14 (50)	4/8 (50)	3/6 (50)	1

Large nerve palsies	2/14 (12)	2/8 (25)	0	0.186

Cerebellum dysfunction	5/14 (38)	2/8 (25)	3/6 (50)	0.334

Laboratory findings				

Indexes of CSF^d ^inflammation				

WBC count (median ± IQR)	550 ± 2400	936 ± 3610	310 ± 520	0.069

< 100 cells/mL	5/40 (12)	3/27 (11)	2/13 (15)	0.702

100-999 cells/mL	20/40 (50)	11/27 (40)	9/13 (70)	0.091

> 999 cells/mL	15/40 (37)	13/27 (49)	2/13 (15)	**0.045**

Protein level,g/L (median ± IQR)	2.4 ± 2.7	2.39 ± 2.26	2.88 ± 3.16	0.357

Protein level > 2 g/L	23/43 (53)	17/29 (67)	6/14 (32)	0.331

% PMN (median ± IQR)	70 ± 59	70 ± 62	65 ± 48	0.418

> 50%	27/40 (67)	19/27 (67)	8/13 (61)	0.576

CSF/blood glucose (median ± IQR)	0.26 ± 0.23	0.27 ± 0.25	0.22 ± 0.23	0.243

CSF Gram stain				

Gram-positive rods	13/43 (30)	9/29 (31)	4/14 (29)	0.869

Positive CSF culture ^e^	40/43 (93)	26/29 (67)	14/14	0.212

4b serotype	23/28 (82)	12/16 (75)	11/12 (92)	0.355

1/2b serotype	5/28 (18)	4/16 (25)	1/12 (8)	0.254

Positive blood cultures	27/43 (63)	18/29 (62)	9/14 (63)	0.643

Blood analytical findings				

WBC count (10^9^/L, (median ± IQR)	12 ± 10	10 ± 11	15 ± 5	0.006

Platelets (10^9^/L, median ± IQR)	182 ± 75	161 ± 87	193 ± 70	0.492

Sodium (mEq/L, median ± IQR)	135.1 ± 4.9	134.2 ± 5	136.9 ± 3.8	0.086

Hyponatremia	19/43 (44)	15/29 (51)	4/14 (28)	0.152

Outcome				

Unfavorable outcome (GOS^f^<4)	15/43 (35)	8/29 (27)	7/14 (50)	0.184

Mortality	12/43 (28)	7/29 (24)	5/14 (35)	0.482

**^a ^**Results are expressed as n (%) unless otherwise specified

**^b ^**Use of at least two doses of an oral or parenteral antibiotic within one week of the onset of meningitis.

^c ^Triad of fever, neck stiffness and change in mental status.

^d ^CSF: Cerebrospinal Fluid.

^e ^The serotype identification only was done in 28 patients.

^f ^GOS: Glasgow Outcome Scale.

In our series, in patients with *Lm *meningitis, fever was the most common symptom (91%). The majority of patients also had altered mental status at presentation, while headache was present in nearly half the patients. The classic triad of fever, neck stiffness and altered mental status was present in 21 (49%) patients; however, almost all patients (95%) had at least 1 or more of these symptoms. Thirty-two percent of the patients had focal neurological findings at presentation, 12% presented cerebellum dysfunction, and 9% had seizures. The most frequent focal neurological finding was cranial nerve palsies. The median duration of symptoms before admission to the hospital was two days. Immunocompromised patients had a lower incidence of the classic triad (45% *vs*. 71%; p = 0.039), headache (59% vs 85%; p = 0.076), and focal neurological deficit at presentation (28% vs 43%; p = 0.317) as compared with immunocompetent patients with acute community-acquired *Lm *meningitis.

Thirty-nine (91%) patients underwent CT in the emergency department. In 30 of 39 (77%), there were no abnormalities associated with the listerial infection. In the remaining 9 (23%) patients, the most common findings were focal lesions and/or hydrocephalus. All patients with hydrocephalus were treated neurosurgically.

Lumbar puncture was performed in all patients. CSF findings are outlined in Table 3. There was no relation between low CSF leukocyte count and age or immunocompromise. The most frequently found serotype was the 4b in 23 (82%) of 28 cases.

Empirical antimicrobial therapy was intravenous ampicillin (2 g every four hours) for 34 (79%) of 43 patients, in 11 (32%) of them combined with aminoglycosides. The rest of six patients received trimethoprim-sulfamethoxazole therapy in two cases and meropenem in the other four cases as empirical antibiotic treatment. Three patients that did not received appropiated empirical treatment receiving ceftriaxone plus vancomycin. Twenty-one patients (49%) received adjunctive therapy with dexamethasone; in 14 cases, the first dose was given previously or concomitantly to the first antibiotic dose, and eleven patients received phenytoin. There were no major side-effects of dexamethasone therapy (gastro-intestinal bleeding, hyperglycemia).

The overall mortality was 28% (12 of 43 patients) and five of thirty-one (16.1%) surviving patients had a neurological deficit at discharge. Neurological abnormalities identified were cranial nerve palsies in 2 (6.4%) and hemiparesis in 3 (9.6%). No patients had hearing impairment. Definitive ampicillin plus gentamicin therapy was significantly associated with unfavourable outcome (67% vs 28%; p = 0.024) and a higher mortality (67% vs 32%; p = 0.040) due to *Lm *infection, Table 4.

**Table 4:** Description of patients with acute community-acquired *Lm *meningitis who survivied or died

	Survivors n = 31	Non-Survivors n = 12	*p*
Age (median ± IQR)	67 ± 26	71 ± 43	0.999

Elderly patient	17 (55)	7 (58)	0.836

Immunocompromised	22 (71)	7 (58)	0.482

Neurological finding	9 (29)	2 (17)	0.698

GCS^b ^at presentation (median ± IQR)	13 ± 11	13 ± 8	0.453

Classic Triad^c^	14 (45)	7 (58)	0.438

WBC > 999 cells/mL	10 (33)	5 (42)	0.282

Protein level g/L (median ± IQR)	2.08 ±1.3	2.1 ± 4.4	0.685

CSF^d^/blood glucose ratio (median ± IQR)	0.27 ± 0.24	0.18 ± 0.21	0.448

Hyponatremia	13 (42)	6 (50)	0.633

Seizures	2 (7)	2 (17)	0.308

Empirical Ampicilllin + gentamicin therapy	8 (26)	3 (25)	0.957

Definitive Ampicillin + gentamicin therapy	10 (32)	8 (67)	**0.040**

Dexamethasone concomitant to antibiotic	17 (55)	4 (33)	0.206

Phenytoin	8 (26)	3 (25)	0.999

**^a ^**Results are expressed as n (%) unless otherwise specified

^b ^GCS: Glasgow Coma Scale.

^c ^Triad of fever, neck stiffness and change in mental status.

^d ^CSF: Cerebrospinal Fluid.

## Discussion

Early administration of adequate antimicrobial therapy is the cornerstone of treatment in patients with meningitis. Despite developments in antimicrobial agents, *Lm *meningitis is still a serious disease that carries high morbidity and mortality rates. A relevant aspect in this study is the prospective inclusion of adult patients with acute community-acquired *Lm *meningitis, to evaluate risk factors associated with *Lm *aetiology which should contribute to early appropriate antimicrobial therapy of this serious and life-threatening disease. Thus, in a large cohort of patients with Ac-ABM, our study reveals that elderly, immunocompromised patients, and a higher CSF/blood glucose ratio were independently associated with isolation of *Lm *as the responsible pathogen.

In our own series, the incidence of acute community-acquired *Lm *meningitis in adults was 16.5% (46/278). Previously, in a multicenter study [14] conducted in the United States, the authors reported that *Lm *accounted for 8% of the cases of bacterial meningitis. Other studies from Europe and North America have reported incidences of 5%-10% or more among all episodes of Ac-ABM among adults [15-18]. Over a 36-year period, Durand *et al. *described that *Lm *accounted for 11% of the episodes of Ac-ABM in adults [7]. The probable increase in the incidence may be attributed to longer life expectancy, changes in diet or food processing, and the higher number and longer survival of immunocompromised people [2].

On the other hand, symptoms and signs of patients presenting with *Lm *meningitis were not different from those found in the general population of patients with Ac-ABM, according to previous studies [19].

The CSF profile in patients with *Listeria *infection revealed significantly fewer WBC and lower protein concentrations than patients with infection due to other pathogens, and a trend towards less hypoglycorrhachia and a lower percentage of PMNs. However, these findings do not apply to all cases [20]. In the appropriate clinical situation, clinicians should not exclude *Lm *based on the degree of pleocytosis, the percentage of PMNs, or the concentration of glucose or protein in the CSF.

The experience in the present series suggests that the Gram stain is negative in two-thirds of the cases of *Lm *meningitis, and may be misleading in many of the remaining cases. Clinicians should be aware of these difficulties, inform the microbiology department when they suspect this pathogen, and not rule out *Lm *based on Gram stain alone. Furthermore, despite the diversity of serotypes of *Lm*, only three serotypes are responsible for >90% of human disease: 1/2a, 1/2b, and 4b. The majority of strains were serotype 4b [21] (23 of 28 cases; 82%), suggesting that serotype 4b of *Lm *is more virulent than others. Otherwise, blood cultures were positive in more than half of the cases in the present series. Some reports advocate the use of the polymerase chain reaction or immunoassays for the early detection of *Lm *[22,23], but these techniques need further evaluation.

Most patients in our case series received appropriated empirical therapy, 40/43 (93%), in 11 of them associated to aminoglycosides. Our study showed that a definitive combined therapy with aminoglycosides was significantly associated with an increasing trend for an unfavourable outcome and mortality, which corresponds with a previous study [24]. These results could be explained by *Lm *meningitis patients have an associated co-morbid condition, in which addition of an aminoglycoside may be harmful. For this reason, actually this combined therapy is been questioned, moreover of the drug's associated nephrotoxicity and inability to cross the blood-brain barrier. In this way, every case should be approached independently, although certain subsets of patients probably require empiric antibiotic treatment for *Lm*, such as patients over 45-50 years of age, patients with immunosuppression, a higher CSF/blood glucose ratio, or patients with a Gram stain of CSF revealing Gram-positive bacilli [19], and the associated with aminoglycosides should be avoid in order to improve patient's outcome.

A significant number of patients, 5 of 31 (16.1%) of the survivors in the present series had some degree of residual neurological deficit at hospital discharge. Although an even higher incidence of residual neurological deficits (32%) was noted among survivors enrolled in 3 previous series and deficits persisted for more than a year in 4 of 10 of those cases with adequate follow up [25-27].

Moreover, mortality due to *Lm *is among the highest of all causes of acute bacterial meningitis [28]. Part of the high mortality of *Listeria *infection may relate to the increased number of immunocompromised and older patients affected by this pathogen [29,30]. Although evidence for use of anticonvulsant prophylaxis in *Lm *meningitis is lacking, it may be considered as seizures have previously been related to increases mortality [31], as did we in the present series, although this was not statistically significant. Further studies are needed in order to clarify this point.

Finally, the role of adjunctive therapy with corticosteroids has been widely debated. A clinical trial showed a beneficial effect of early dexamethasone treatment in adults with bacterial meningitis [32]. Nevertheless, there are no studies on the effect of dexamethasone in adult *Listeria *meningitis. We observed a higher survival rate among those patients that received dexamethasone concomitant to antibiotic, although this association did not reach statistical significance. Our results suggest that further studies are needed to evaluate the effect of corticosteroid therapy in adult patients with *Lm *meningitis.

The present study has several limitations. First, given the small number of patients, we were unable to develop a statistical model to identify risk factors independently associated with a poor outcome or death in patients with acute community-acquired *Lm *meningitis. Secondly, only patients who underwent lumbar puncture and who had a positive cerebrospinal fluid culture were included. Negative cerebrospinal fluid cultures occur in 20 percent of patients with acute community acquired bacterial meningitis. Third, delay of antimicrobial therapy was not recorded. Despite all these limitations, we consider that this study contributes to our knowledge of risk factors associated with development of acute community-acquired *Lm *meningitis, and to evaluating epidemiology, clinical features, management, and outcome in this homogenous population of adult patients with acute community-acquired *Lm *meningitis.

## Conclusions

To sum up, our study showed elderly or immunocompromised patients and a higher CSF/blood glucose ratio in patients with Ac-ABM must alert clinicians about *Lm *aetiology. Furthermore, we observed a high incidence of acute community-acquired *Lm *meningitis in adults and the addition of aminoglycosides to treatment should be avoid in order to improve the patients' outcome. Nevertheless, despite developments in intensive care and antimicrobial therapy, this entity is still a serious disease that carries high morbidity and mortality rates. Further studies should focus on specific interventions (e.g. adjuvant therapies) that could help to improve the poor prognosis of these patients.

## Competing interests

The authors declare that they have no competing interests.

## Authors' contributions

RAV conceived the study and participated in its design and coordination and helped draft the manuscript. EGC carried out the acquisition of data and performed the statistical analysis. ESI participated in the acquisition, analysis and interpretation of data. PFV participated in the design of the study, acquisition of data and drafting of the manuscript. DFA participated in the conception, design and coordination of the study. PCA participated in the coordination and helped to draft the manuscript. CRG participated in the conception of the study and helped to draft the manuscript. ACC participated in the coordination and helped to draft the manuscript. FGD participated in the conception, design and coordination of the study. AGE participated in the coordination and helped to draft the manuscript. JP conceived the study and participated in its design and coordination and helped to draft the manuscript. GPP conceived the study and participated in its design and coordination. All authors read and approved the final manuscript.

## Pre-publication history

The pre-publication history for this paper can be accessed here:

http://www.biomedcentral.com/1471-2334/10/324/prepub
